# Influence of Free and Microencapsulated Extracts from Onion Peels on the Performance of Fortified Fresh Cheese

**DOI:** 10.3390/foods15061048

**Published:** 2026-03-17

**Authors:** Sara M. Ferreira, Lúcia Santos

**Affiliations:** 1 LEPABE—Laboratory for Process Engineering, Environment, Biotechnology and Energy, Faculty of Engineering, University of Porto, Rua Dr. Roberto Frias, 4200-465 Porto, Portugal; 2 ALiCE—Associate Laboratory in Chemical Engineering, Faculty of Engineering, University of Porto, Rua Dr. Roberto Frias, 4200-465 Porto, Portugal

**Keywords:** onion peels, phenolic compounds, microencapsulation, functional dairy products, phenolic stability

## Abstract

As a strategy to valorise onion peel (OP), a phenolic-rich extract was obtained and microencapsulated using the double emulsion technique for improved stability. Both free and microencapsulated OP extracts were added to fresh cheese to enhance its nutritional composition. The extract exhibited a high total phenolic content (TPC) and strong antioxidant capacity towards ABTS and DPPH radicals, with IC_50_ of 9.5 and 36.1 mg_Extract_∙L^−1^, respectively. The extract demonstrated inhibitory capacities of 71% against α-amylase and 82% towards β-glucosidase. Quercetin was identified as the main phenolic compound, while potassium was the predominant mineral. The microencapsulation yielded an encapsulation efficiency of 91%, with an average particle size of 17.9 µm. Incorporating free and microencapsulated OP extract into the fresh cheese reduced syneresis, a favourable outcome, while preserving moisture levels, protein and ash content, and the pH. The incorporation of the free and microencapsulated OP extract enhanced the TPC and DPPH scavenging capacity of the cheeses. Results demonstrated the potential of using OP extract to enhance the antioxidant properties of fresh cheese, and to reduce syneresis, while promoting sustainability. These outcomes are particularly relevant from an industrial point of view, since an increase in antioxidant content might contribute to extending the product shelf-life.

## 1. Introduction

Antioxidants are compounds that have been gaining attention in modern society over the past few decades. An antioxidant is defined as a compound, synthetic or natural, that is capable of delaying or preventing the oxidation of a substrate when added to products [1,2]. Moreover, these compounds can scavenge reactive oxygen species (ROS) and reactive nitrogen species (RNS), preventing their development, and can break chains of free radical propagation [1]. ROS and RNS are by-products of cellular redox reactions and play a crucial role in human body biochemistry. However, at high concentrations, these radicals can lead to oxidative stress, affecting the structure and function of cells, triggering negative repercussions for human health, including neurological and cardiovascular problems [3]. Additionally, the constant exposure of humans to pollution, chemicals, cigarettes, stress, and other factors in today’s world increases oxidative stress. Consequently, antioxidants have been gaining attention due to their potential to surpass these effects. Therefore, consumers have been seeking offers to enhance their regular intake of antioxidants to prevent oxidative stress [4].

Antioxidants are crucial in food preservation since they delay the oxidation of certain nutrients, such as lipids and sugars, which can decrease the nutritional value of foods and produce unpleasant smells [5]. Additionally, they contribute to maintaining the organoleptic properties and prevent food deterioration [5,6]. These compounds are essential for extending food shelf life and maintaining its quality throughout storage and consumption phases. In the dairy industry, multiple antioxidants can be used, including sorbic acid, potassium sorbate, butylated hydroxyanisole (BHA), butylated hydroxytoluene (BHT), propyl gallate (PG), and tertiary butylhydroquinone (TBHQ), depending on the legislation of each country [7,8,9]. However, scientific research has shown that regular consumption of some of these compounds might lead to potential health problems, including cancer and cytotoxic effects. In fact, research has shown that BHA has low acute toxicity in rats, with a lethal dose superior to 2000 mg∙kg^−1^; however, it was reported that this compound has strong cytotoxicity on human astrocytes, while BHT has been reported to have cardiotoxic effects and might be a teratogen for aquatic organisms [10]. Consequently, the investigation for healthier and more sustainable alternatives has increased over the past years [7,9,11].

Onions are a major vegetable consumed worldwide due to their interesting nutritional composition and rich content of bioactive compounds, including phenolic compounds [12]. According to the FAO (Food and Agriculture Organisation of the United Nations), the world production of this vegetable in 2024 was approximately 108.3 million tons [13]. Onion bulbs are the primary part consumed, while the rest of the onion is discarded, resulting in high amounts of by-products, mainly onion peel (OP), which account for approximately 60% of the produced by-products [14]. Fortunately, OP are a rich source of bioactive compounds, including organosulfur compounds, a wide variety of phenolic compounds (polyphenols and flavonoids), vitamins, among others [12,14,15,16]. Furthermore, OP contains significant concentrations of essential minerals such as calcium, potassium, and magnesium [15]. These inorganic compounds play an important role in various biochemical functions inthe human body [17]. Phenolic compounds are known for their biological properties and beneficial effects for human health. Literature reveals that these compounds exhibit antioxidant, antibacterial, anticarcinogenic, anti-diabetic and anti-inflammatory properties, which can have therapeutic effects for humans after consumption [18,19]. A primary positive aspect of phenolic compounds on human health is the reduction in oxidative stress; moreover, they might help manage cardiovascular and neurological disorders, hypertension, inflammation, obesity, and infections, among others [20,21,22]. As a result, OP have been gaining attention as they display promising potential as functional ingredients in the food industry, since they may act as natural antioxidants, preservatives and additives. In fact, OP can be used as raw material to obtain phenolic-rich extracts that can later be incorporated into different foods, enhancing their nutritional value.

Despite their potential as functional ingredients, the incorporation of phenolic compounds into foods poses some limitations. The main one is their limited stability, as they can be easily degraded by external factors, including oxygen, pH, light, temperature, enzymatic activity, and interactions with other food components [23]. Another limitation is their low bioavailability (amount of compounds available for absorption through the bloodstream and/or lymphatic systems) and bioaccessibility, which is the amount of compound released from the food matrix into the gastrointestinal tract and is available for absorption [24,25]. Furthermore, certain phenolic compounds, such as quercetin and anthocyanins, exhibit colour while also possessing astringent and bitter characteristics [23,26]. This can result in undesirable alterations in the taste and colouration of the product. Microencapsulation is one approach that can be employed to overcome the limitation of incorporating free phenolic compounds into foods. It is a combination of techniques that ensures that the desired compounds are embedded in a polymeric matrix, commonly designated as the encapsulating agent [23]. This tactic helps to protect phenolic compounds, guaranteeing their biological potential; moreover, it allows the controlled release of compounds and can enhance their bioaccessibility during gastrointestinal digestion. Double emulsions are defined as complex systems in which the active compound is encapsulated in small quantities within droplets of a dispersed oil phase, which is dispersed in an aqueous phase. While a variety of techniques have been employed to encapsulate phenolic compounds (both for standard compounds and extracts derived from plants and agro-industrial by-products), this method exhibits significant potential for achieving enhanced encapsulation efficiencies for these compounds [23,26,27,28,29,30].

Fresh cheese is a typically consumed cheese due to its mild flavour, along with its interesting composition, since it displays high protein content, vitamins and minerals. However, this dairy product presents a major limitation regarding its low shelf life, as its pH and high moisture content provide optimal conditions for pathogenic growth and, consequently, microbial spoilage [31,32]. Moreover, this product is susceptible to oxidation due to its fat content, which can lead to the development of unpleasant flavours and odours, and a potential decrease in nutritional content. The combination of these factors reduces the shelf life of fresh cheese, affecting the production, shipping, and selling process of the product. To minimise this problem, preservatives and antioxidants can be incorporated into fresh cheese. As a strategy to decrease the use of synthetic and commercial antioxidants/preservatives, phenolic compounds from plants and agro-industrial by-products can be used to fortify fresh cheese. This approach is in line with literature trends, which have been focused on the fortification of various dairy products and cheeses using different extracts (rich in vitamins, phenolic compounds, antioxidants) from plants and by-products [33,34]. Literature indicates that cheese fortification is an emerging trend within the scientific community. However, current studies predominantly focuson the incorporation of natural extracts, typically examining them in their unencapsulated form [35,36,37,38]. This leaves a notable gap in the understanding of the integration of extracts derived from by-products, as well as the incorporation of microparticles into the cheese matrix. 

The present study aimed to evaluate and compare the fortification of fresh cheese with free and microencapsulated phenolic-rich extracts from onion peels. The impact of varying microparticle quantities was evaluated to determine how this parameter affects the performance of the cheese. The phenolic extract was characterised regarding its antioxidant activity towards ABTS, DPPH and biological radicals and potential anti-diabetic capacities, along with its chemical and mineral content and its minimum inhibitory concentration against *Escherichia coli* and *Staphylococcus aureus* was evaluated. To understand the effect of the free and microencapsulated phenolic extract on fresh cheese performance, the organoleptic, physicochemical and antioxidant properties were assessed, along with the proximate and mineral composition of the cheeses. In contrast to the author’s previous study, this research aimed to assess the effects of OP extract (both free and microencapsulated) on cheese with high fat content that is rennet-coagulated, utilising proteolytic enzymes instead of acid-coagulation, assessing its effects within a more structured matrix.

## 2. Materials and Methods

### 2.1. Chemicals and Reagents

Local farmers from the district of Porto, North of Portugal, provided onion peel (OP). Pasteurised milk was purchased in a local supermarket. Ethanol, used as the extraction solvent, was obtained from VWR International (Radnor, PA, USA). The reagents used for extract characterisation, 2,2-diphenyl-1-picrylhydrazyl (DPPH), 2,2′-azino-bis (3-ethylbenzothiazoline-6-sulfonic acid) (ABTS), and *Folin–Ciocalteu* reagent, were sourced from Sigma Aldrich (St. Louis, MO, USA), and sodium carbonate was purchased from Merck (Darmstadt, Germany). For the microencapsulation of the extract, ethyl cellulose (Ref. 433837) and polyvinyl alcohol (Ref. P8136) were acquired from Sigma-Aldrich, while ethyl acetate was purchased from VWR International. Ultrapure water (Ref. 83645.320, H_2_O, CAS 7732-18-5) was obtained from VWR International.

### 2.2. Phenolic-Rich Extract Obtainment and Characterisation

To obtain a phenolic-rich extract from OP, a pre-treatment of the raw material was conducted. Initially, peels exhibiting any signs of degradation were removed, while the intact peels were cleaned. Subsequently, the OP were subjected to drying in an oven at 60 °C for 16 h to facilitate moisture removal. Following this drying process, the sample was ground using a coffee grinder to achieve a uniform particle size, ensuring homogeneity for extraction. The extract was then obtained through a solid–liquid extraction process using a Soxhlet apparatus, with ethanol serving as the extraction solvent. The extraction was carried out at a sample-to-solvent ratio of 1:20 (m/V) for 2 h. Soxhlet extraction was selected, instead of greener techniques, due to its effectiveness in extracting phenolic compounds, yielding notable extraction results. Additionally, the group has already optimised the extraction conditions, further enhancing its efficiency. To ensure complete removal of the solvent, the solution was subjected to rotary evaporation followed by a nitrogen stream.

The antioxidant properties of the OP extract were evaluated by analysing the Total Phenolic Content (TPC) and the extract’s inhibitory capacity towards the ABTS and DPPH radicals. TPC was determined employing the methodology with the *Folin–Ciocalteu* reagent, according to the literature [39]. The results were expressed in milligrams of gallic acid equivalents (GAE) per gram of OP extract, derived from the calibration curve: Abs = 0.0818·*C*(mg_GAE_·L^−1^)—0.023. The assays for the ABTS and DPPH radicals were carried out using optimised protocols previously established by the authors’ research group [39].

The performance of the OP extract to scavenge biological radicals was evaluated using assays with the superoxide anion radical (O_2_^−•^) and the nitric oxide radical (•NO), according to literature protocols [40,41]. For the superoxide scavenging assay, an OP extract solution (c = 6 mg∙mL^−1^) was prepared in DMSO. Dilutions up to 0.05 mg∙mL^−1^ were prepared in 60% DMSO in KH_2_PO_4_ buffer. In a 96-well microplate, 50 µL of the extract solutions were mixed with 150 µL NBT (nitrotetrazolium blue chloride, 43 µM) and 50 µL of NADH solution (β-nicotinamide adenine dinucleotide reduced form, 166 µM in KH_2_PO_4_ buffer, pH 7.4). Afterwards, 50 µL of PMS solution (phenazine methosulphate, 2.7 µM) were added to each well to start the reaction, and the absorbance was read at 562 nm, in kinetic mode for 2 min. The results were expressed in O_2_^−•^ scavenging percentage. For the nitric oxide scavenging assay, the Griess reaction was used. A solution of OP extract (c = 2 mg∙mL^−1^) was prepared in DMSO. The sample was diluted up to 0.06 mg∙mL^−1^ in 20% DMSO in KH_2_PO_4_ buffer (100 mM, pH = 7). Afterwards, 75 µL of the samples were added to a 96-well microplate with 75 µL of SNP (sodium nitroprusside—Na_2_[Fe(CN)_5_NO]·2H_2_O, 2.5 mg·mL^−1^ in KH_2_PO_4_ buffer), which was used as •NO donor. The microplate was left under light for 60 min, at room temperature. Then, 75 µL of Griess reagent (1% sulphanilamide and 0.1% *N*-(1-napthyl) ethylenediamine chloride in 2% H_3_PO_4_). The absorbance was read at 562 nm, after 10 min of incubation in the dark. The results were expressed in •NO scavenging percentage.

The anti-diabetic properties of the extract were determined using inhibition assays towards α-amylase and β-glucosidase. The α-amylase inhibition assay was performed using a protocol from the literature [39]. For the β-glucosidase inhibition assay, the Sigma-Aldrich β-glucosidase Activity Assay Kit (MAK129) (Sigma Aldrich, St. Louis, MO, USA) was used. The percentage of enzyme inhibition was calculated by comparing the enzyme activity in the absence of the inhibitor to its activity in the presence of the inhibitory agent (OP extract).

The minimum inhibitory concentration (MIC) test of the extract was performed to assess its antibacterial activity towards *Escherichia coli* and *Staphylococcus aureus*. This parameter was evaluated using the broth microdilution method in a sterile 96-well microplate, with Mueller-Hinton (MH) broth, according to literature [42,43]. To do so, bacterial strains, at −80 °C, were dispersed onto PCA (plate count agar) plates and incubated for 24 h, at 37 °C. The inoculum was prepared by transferring a bacterial colony into 25 mL of MH broth; the inoculum was incubated overnight with continuous shaking at 120 rpm at 37 °C. Afterwards, the bacterial cells were collected, and the cell density was adjusted to a concentration of 1 × 10^6^ cells∙mL^−1^, at an OD of 0.132 ± 0.02 at 600 nm. The assay was performed by adding 20 µL of extract solution (six concentrations were evaluated: 10,000, 7500, 5000, 2500, 1000 and 500 µg∙mL^−1^) to a sterile 96-well polystyrene microplate, followed by 180 µL of bacteria cells. A solution of UPW with 5% DMSO was used as a negative control. Additionally, 180 µL of bacteria cells were mixed with 20 µL of MH broth to evaluate bacterial growth under normal conditions. The plates were incubated for 24 h at 37 °C. The OD was measured before and after the incubation period. MIC was defined as the lowest concentration of OP extract that prevents microorganism growth, which means when the final OD was equal to or inferior to the initial OD [42].

The mineral composition of OP powder and OP extract was determined by microwave-assisted digestion, followed by ICP-OES (Inductively Coupled Plasma Optical-Emission Spectrometry) analysis. For the microwave digestion, 0.1 g of the sample were mixed with 6 mL of HNO_3_ 65% and 2 mL of H_2_O_2_ 30% in a digestion vessel. Microwave-assisted digestion was carried out using a Milestone START D Microwave Digestion System (Sorisole, Italy). The programme consisted of a 15 min temperature ramp to 200 °C, a 15 min hold at 200 °C, followed by a 15 min controlled cooling ramp to 110 °C, and a final 25 min ventilation step for cooling. The analysis was performed in triplicate. Afterwards, the digested samples were filtered using a 0.2 μm filter, and their volume was adjusted to 25 mL with ultrapure water (UPW) to proceed with ICP analysis. The analysis was performed in a Thermo Scientific^®^ iCAP 7400 ICP-OES Duo, coupled with a CETAC^®^ ASX-520 Autosampler, with argon as plasma source.

### 2.3. Microparticles Production

The OP extract was microencapsulated using the water-in-oil-in-water (w/o/w) double emulsion solvent evaporation technique. A theoretical loading of 10% (m/m) was used. The primary aqueous phase was prepared by dissolving the OP extract in ultrapure water (UPW), according to the desired loading. The oil phase was prepared by dissolving 200 mg of ethyl cellulose in 10 mL of ethyl acetate to form the polymer solution, which was vortexed for 15 min. The primary emulsion was prepared by adding 1 mL of the primary aqueous phase to the oil phase, and the mixture was vortexed for 5 min. Afterwards, the emulsion was added to a solution of poly(vinyl alcohol) (PVA) (1% m/V) and emulsified using a high-performance homogeniser at 5000 rpm for 5 min, creating the double emulsion. This solution was then mixed on a stirring plate at 700 rpm for 4 h in the fume hood at room temperature to evaporate the solvent. Then, the microparticles were recovered by filtration using a 0.2 μm filter and washed with UPW to remove any residual PVA. The microparticles were then frozen for 24 h at −80 °C and then lyophilised for 24 h.

### 2.4. Microparticles Characterisation

The produced microparticles were characterised regarding production yield (PY), encapsulation efficiency (EE) and actual loading content (ALC), using Equations (1)–(3), respectively.

(1) $$\mathrm{PY}\;=\frac{m_{\mathrm{MP}}}{m_{H}+m_{\mathrm{polymer}}}\;\times 100\;$$

(2) $$\mathrm{EE}\;=\frac{m_{\mathrm{BAC},\;\mathrm{encaps}}}{m_{H}}\;\times 100=\frac{m_{H}-m_{S}}{m_{H}}\;\times 100$$

(3) $$\mathrm{ALC}\;=\frac{m_{\mathrm{BAC},\;\mathrm{encaps}}}{m_{\mathrm{MP}}}\;\times 100$$

where *m*_MP_ is the mass of the lyophilised OP extract-loaded microparticles, and *m*_H_ is the mass of extract initially added. The *m*_BAC,encaps_ was calculated considering the difference between *m*_H_ and the mass of extract present on the surface of the microparticles (*m*_S_). To quantify the mass of extract present on the surface (*m*_S_), a solution of 1000 mg∙L^−1^ of microparticles in ethanol was prepared, and the TPC of the sample was calculated following the methodology described in Section 2.2.

### 2.5. Particle Size Distribution and Polydispersity

A Coulter Counter-LS 230 Particle Size Analyzer (Miami, FL, USA) was used to determine the particle size distribution of the produced microparticles, using laser granulometry. The differential volume distribution was considered during the measurement of the particle size distribution. Additionally, the polydispersity index (PDI) was described according to the literature [27].

### 2.6. Fourier Transform Infrared Spectroscopy (FTIR)

To understand if there were any interactions between the polymer and the OP extract and evaluate if the extract was successfully embedded in the polymer, the FTIR spectra of the samples were obtained using a FTIR-ATR spectrometer (Spectrum Two, PerkinElmer, Waltham, MA, USA). The mode of attenuated total reflectance (ATR) was used to measure samples, using an A225/Q PLATINUM ATR Diamond crystal with a single reflection accessory.

### 2.7. Differential Scanning Calorimetry Analysis

To assess the thermal behaviour of the OP extract-loaded microparticles, OP extract, unloaded microparticles and ethyl cellulose, the Differential Scanning Calorimetry (DSC) technique was employed. The analyses were performed with a temperature range of −20 °C to 250 °C, at a heating rate of 10 K∙min^−1^, under a nitrogen atmosphere, using a NETZSCH DSC 214 Polyma calorimeter (NETZSCH DSC 214 Polyma, Selb, Germany).

### 2.8. Morphological Analysis

A Phenom ProX Desktop SEM (Thermo Fisher Scientific, Waltham, MA, USA) scanning light microscope, at an accelerating voltage of 10 kV, was used to evaluate the morphology of the OP extract-loaded microparticles and the polydispersity of the sample.

### 2.9. Fresh Cheese Production

To produce the fresh cheese, 1 L of pasteurised cow whole milk was warmed up to 38 °C. After reaching this temperature, 1 g of NaCl and 1 mL of rennet were added to the milk and stirred. The solution was left to incubate at room temperature for 30 min. Subsequently, the mixture was filtered to separate the cheese from the serum and placed in a mould. Afterwards, the cheese was stored in a refrigerator, at 4 °C, until further use. Six cheeses were produced: NC: Negative control (without additives), OP: Cheese with 1 g∙L^−1^ of OP extract, Un: Cheese with 1 g∙L^−1^ of unloaded microparticles, MP 1: Cheese with 1 g∙L^−1^ of OP extract loaded microparticles, MP 2: Cheese with 2.5 g∙L^−1^ of OP extract loaded microparticles, MP 3: Cheese with 5 g∙L^−1^ of OP extract loaded microparticles. The objective of this study was to evaluate the impact of varying microparticle concentrations on the targeted outcomes. The selected concentrations were determined as follows:

- MP 1: This formulation had a microparticle concentration of 1 g∙L^−1^, corresponding to the same concentration of the OP extract, to assess the direct effect of microparticles at this specific dosage.
- MP 2: An intermediate concentration was utilised to explore the effects of microparticles at levels between the OP extract and those of higher concentrations.
- MP 3: This formulation involved the application of microparticles at 5 g∙L^−1^, which contained 0.5 g of OP extract. It is noteworthy that higher concentrations, such as 10 g∙L^−1^ of microparticles, were deliberately excluded from the evaluation, as such levels may adversely affect the physical properties of the cheese due to the presence of polymer within the microparticles.

Formulation Un was developed to investigate the influence of the encapsulating agent, ethyl cellulose, on the results. The cheeses were subjected to stability tests at five testing times: t_1_—1 storage day; t_2_—4 storage days; t_3_—7 storage days; t_4_—10 storage days; t_5_—14 storage days. Typically, fresh cheese has a limited shelf life (from 7 to 10 days); thus, to evaluate the potential to extend it, the present study was conducted over a fortnight.

### 2.10. Determination of the Proximate and Mineral Composition of the Cheese

To determine the proximate composition of the produced fresh cheeses, the protein, fat and ash content were determined. The protein content was calculated by the determination of the total nitrogen using the Kjeldahl method, and a conversion factor of 6.38 was used to express the results as a percentage of protein, according to the AOAC standard method [44]. The fat content was determined using the Folch method [45]. For the ash content, the determination was performed according to the ISO 6884 guidelines.

The determination of the mineral composition of the samples was performed according to the procedure described in Section 2.2, for the analysis of the mineral composition of OP and OP extract.

### 2.11. Physicochemical Characterisation of the Fresh Cheese

The syneresis of the samples was evaluated by weighing 2 g of each sample. The samples were then centrifuged for 20 min at 700× *g*, using a Rotofix 32 A Centrifuge (Hettich, Germany). Afterwards, the mass of supernatant was measured, and syneresis was calculated according to Equation (4):

(4) $$\mathrm{Syneresis}\;(\%)=\;\frac{m_{\mathrm{supernatant}}}{m_{\mathrm{cheese}}}\;\times 100$$

The determination of the moisture content of the samples was performed by weighing approximately 1 g of the sample. The sample was then left to dry at 105 °C until a constant mass was achieved. The moisture content was determined using Equation (5):

(5) $$\mathrm{Moisture}\;\mathrm{content}\;(\%)=\;\frac{{m_{\mathrm{cheese}}-m}_{\mathrm{dried}\;\mathrm{cheese}}}{m_{\mathrm{cheese}}}\;\times 100$$

The pH value of the samples was determined by adding 9 mL of water to 1 g of sample and mixing it using a high-performance homogeniser (IKA T18 digital Ultra Turrax) for 1 min. The pH of the solution was then measured with a digital pH metre (XS pH 50+).

### 2.12. Total Phenolic Content and Antioxidant Capacity of the Fresh Cheese

In order to determine the TPC and the antioxidant capacity of each fresh cheese sample, phenolic compounds were extracted from the samples. For that, 2 g of each sample were mixed with 4 mL of ethanol using a vortex (VWR VV3) for 1 min; then, the solutions were placed in an ultrasonic bath for 5 min. The process was repeated three times, and after the last one, the sample was centrifuged at 1510× *g* for 10 min. The supernatant was collected and added to a different tube. Afterwards, 4 mL of ethanol were added to the initial sample and the process was repeated. The supernatant was collected and added to the previous one. The samples were mixed and centrifuged once more. The TPC and DPPH scavenging capacity of the samples were determined following the protocols described in Section 2.2.

### 2.13. Statistical Analysis

To determine statistically significant differences between results, *p* < 0.05 (95% confidence interval), the analysis of variance (2-way-ANOVA) was performed, using the GraphPad (GraphPad Prism 8.0) software. The null hypothesis is correct when all the sample values are the same or do not significantly differ from one another. Post hoc comparisons were performed using Tukey’s HSD test. Additionally, before the ANOVA analysis, the normality of the datasets was assessed using the Shapiro–Wilk test.

## 3. Results and Discussion

### 3.1. Phenolics Extraction from Onion Peel and Characterisation

Solid–liquid extraction, with a Soxhlet apparatus, was used to obtain the phenolic-rich extract from OP. Afterwards, the extract was characterised considering its biological properties, including total phenolic content, ABTS, DPPH, superoxide anion radical (O_2_^−•^) and the nitric oxide radical (•NO) scavenging capacity and the inhibition of α-amylase and β-glucosidase. Moreover, the chemical composition of the extract was obtained using HPLC-DAD, and the mineral composition was determined using ICP-OES. The characterisation results are displayed in Table 1.

From Table 1, it can be observed that the TPC of the OP extract was 513 mg_GAE_∙g_extract_^−1^. Comparing with literature, it can be observed that the value achieved is within the typical range, more towards the superior end. A study focusing on the extraction of bioactive compounds from OP using maceration and ethanol as solvent revealed that the TPC was 331 mg_GAE_∙g_extract_^−1^; moreover, the study demonstrated that the IC_50_ of the extract towards the DPPH radical was 22.4 mg_Extract_∙L^−1^ [46]. Another study evaluated the influence of using different extraction techniques on OP, indicating that supercritical extraction leads to a superior TPC of 213 mg_GAE_∙g_extract_^−1^, while conventional solid–liquid extraction, with 70% ethanol and 12.5% of glacial acetic acid, led to a TPC of 150 mg_GAE_∙g_extract_^−1^ [47]. In other studies by the authors, using the same extraction conditions, the obtained TPC values were around 360–400 mg_GAE_∙g_extract_^−1^ [26,27]. Overall, the result indicates that the extract has a high reducing capacity of the *Folin–Ciocalteu* reagent, potentially due to the presence of phenolic compounds [48]. Based on the radical-scavenging assays, the OP extract demonstrated superior effectiveness against the ABTS radical than against the DPPH radical. This result was anticipated since the ABTS radical is soluble in aqueous and organic media, while DPPH is only soluble in organic media [49]. As a result, ABTS might be more effective in quantifying the scavenging capacity of phenolic extracts, since it can screen lipophilic and hydrophilic samples, as is the case with phenolic compounds. Nonetheless, it demonstrated significant inhibitory effects on both types of radicals, highlighting its potential as a radical scavenger. Indeed, it can be observed that the IC_50_ (needed concentration to inhibit 50% of radicals) is inferior to 50 mg_Extract_∙L^−1^, revealing that the extract demonstrates strong antioxidant properties, as described in the literature [50,51]. Moreover, the extract exhibits similar performance to Trolox (a reference antioxidant) in both assays, displaying values of 307 mg_Trolox_∙g_extract_^−1^ and 222 mg_Trolox_∙g_extract_^−1^ for ABTS and DPPH, respectively. It was anticipated that the extract possessed a high TPC and elevated antioxidant potential, since previous studies demonstrated that the OP extract was rich in phenolic compounds, including quercetin [27,39]. These molecules are known for their capacity to scavenge free radicals due to the hydroxyl groups in their structure and their capacity to donate hydrogens to stabilise free radicals, forming the phenoxy radical that is stabilised through resonance.

To better understand the potential of the OP extract in human health, scavenging assays towards the superoxide anion radical (O_2_^−•^) and the nitric oxide radical (•NO) were performed. The superoxide anion radical is the main ROS formed and is a precursor of most ROS. This radical is formed enzymatically, via auto-oxidation, and during respiration, and is mainly produced in the mitochondria [49,52,53]. Despite being essential to human biochemistry, this anion may generate other free radicals that can induce cell damage [54]. Nitric oxide radical is one of the RNS, and is crucial for human biochemistry, mainly in neurotransmission, vascular regulation and immune responses, at low concentrations. However, at high concentrations, this radical can lead to health problems, including inflammation [52,53]. Analysing the results, it can be noted that, at a concentration of 0.5 mg_Extract_∙mL^−1^, the inhibition percentage of these radicals is 98.6% for the superoxide radical and 41.9% for the nitric oxide. Moreover, the IC_50_ for these radicals was 91.5 and 282 mg_Extract_∙L^−1^, for the superoxide and the nitric oxide, respectively. It can be concluded that the OP extract displays considerably superior scavenging capacity towards the superoxide radical than to the nitric oxide radical. A literature study revealed that an OP extract obtained with ethyl acetate, at a concentration of 500 µg∙mL^−1^ (equivalent to 0.5 mg∙mL^−1^), inhibited approximately 97% of superoxide anion radical. Additionally, at the same concentration, the extract inhibited 93% of nitric oxide radicals [54]. In another study, the OP extract was obtained with ethanol:water (70:30). Results revealed that the extract was significantly more active towards the superoxide radical than nitric oxide, displaying IC_50_ values of 56 mg_Extract_∙L^−1^ for the nitric oxide radical and 27 mg_Extract_∙L^−1^ for the superoxide one [55]. Another study observed a similar trend in which the OP extract was more effective at scavenging the superoxide radical. Moreover, the authors achieved an IC_50_ of 112 mg_Extract_∙L^−1^ for the superoxide radical; for nitric oxide, since the scavenging activity was inferior to 50%, this value was not determined [56]. The results achieved are different from the literature ones, which may be attributed to the use of different onion varieties, growing conditions and extraction techniques. Nevertheless, the obtained OP extract exhibited similar performance to the literature, being more effective towards the superoxide radical than nitric oxide. Hence, it can be concluded that the OP extract displays activity towards biological radicals. This activity might be associated with the presence of quercetin, since this phenolic compound is known for its intense scavenging and preventing capacity towards multiple radicals, mainly the superoxide anion and nitric oxide radicals [56].

The potential anti-diabetic effects of OP extract were evaluated by examining its inhibitory activity against the enzymes α-amylase and β-glucosidase. In managing type 2 diabetes, a key strategy involves inhibiting these enzymes, as this can effectively reduce the breakdown of polysaccharides and limit glucose absorption in the digestive tract [57]. By slowing down these processes, OP extract may play a significant role in regulating blood sugar levels and supporting diabetes management. It can be concluded that the OP extract inhibited approximately 71% and 82% of α-amylase and β-glucosidase, respectively, demonstrating a high inhibitory effect, especially towards β-glucosidase. Considering α-amylase, the literature reports that the inhibitory effect of the OP extract is typically between 60–90%, demonstrating that the achieved value is within the typical range [26,58,59]. Regarding β-glucosidase inhibition, the literature is scarce; however, a study performed by the authors revealed an inhibitory effect of approximately 86% of the OP extract towards this enzyme [26]. The difference between results might be associated with the use of different onion varieties and growing conditions, as these aspects greatly impact the composition of bioactive compounds on the peels. Nonetheless, the results indicate the potential anti-diabetic effects of the OP extract in inhibiting α-amylase and β-glucosidase.

Considering the chemical composition of the OP extract, analysis using HPLC-DAD revealed that quercetin was the main phenolic compound present in the extract in a concentration of 58 mg_compound_∙g^−1^. The analysis also demonstrated that the extract exhibited interesting concentrations of epicatechin, followed by resveratrol and kaempferol. Onion peels have been described in the literature as a rich source of quercetin, confirming the achieved results [39,60]. In another study by the authors, performed under similar conditions, quercetin was also found to be the main compound, followed by resveratrol and kaempferol. Therefore, the obtained results are within literature values.

To deepen the knowledge regarding the OP extract characterisation, the MIC against *E. coli* and *S. aureus* was determined. These bacteria were selected since they are Gram-negative and Gram-positive, respectively, and are commonly responsible for human infections. The MIC is defined as the minimum concentration in the microplate at which no visible bacterial growth occurs after 24 h of incubation with consistent agitation and temperature conditions. The achieved results revealed that the concentrations used in the study did not inhibit the growth of *E. coli* and *S. aureus*, suggesting that the obtained OP extract displays low antibacterial activity. However, there was a notable trend indicating that the OP extract could delay bacterial growth at a concentration of 500 µg∙mL^−1^. A literature study reported that the MIC of extracts from OP varies with the extraction solvent, revealing that an ethanolic OP extract was capable of inhibiting the growth of *E. coli* and *S. aureus* at a concentration of 400 and 200 µg∙mL^−1^, respectively [46]. The observed difference suggests variability in the composition of the extracts, indicating that the obtained OP extract may possess a reduced concentration of compounds with antibacterial properties. This finding underscores the influence of onion variety and cultivation conditions on the biological properties of the resulting extracts.

Literature indicates that OP are abundant in minerals; however, there is limited information regarding the mineral composition of phenolic extracts derived from OP. Therefore, to assess the mineral richness of the OP, an ICP-OES analysis was conducted for both the peels and the extract. This analysis aimed to determine whether the extraction process affected the mineral composition of the resultant extract. From the results, the predominant minerals found in OP were calcium (20.9 mg∙g^−1^), followed by potassium (7.3 mg∙g^−1^), sodium (≈2.8 mg∙g^−1^) and magnesium (≈2.7 mg∙g^−1^). Other minerals, including aluminium and iron, were also detected in lower concentrations. In fact, the literature suggests that calcium is the primary mineral found in onion peels, often reported in concentrations of 2000 µg∙g^−1^, along with magnesium and potassium [61,62]. A study evaluating the mineral composition of different parts of the onion, using ICP-MS, revealed that potassium was the primary mineral found (≈16 mg∙g^−1^), followed by calcium and magnesium [63]. It is worth noting that the discrepancies between the findings of this study and those reported in the literature may be due to the use of different onion varieties, variations in cultivation conditions, and differences in the analysis methodology.. Nevertheless, despite these variations, the results indicate that OP are rich in minerals, highlighting their potential for use in food applications. Considering the composition of the OP extract, it can be concluded that the potassium, sodium, and calcium were the main minerals found in the extract in concentrations of 374 µg∙g^−1^, 254 µg∙g^−1^ and 187 µg∙g^−1^, respectively. Apart from boron and strontium, the minerals present in OP were also detected in the OP extract, although in lower concentrations. It was expected that the concentration of minerals in the extract would be lower, potentially undetectable (as happens with boron and strontium), compared to that in the OP powder. This expectation is attributed to the use of ethanol as the extraction solvent, which is not typically regarded as the most effective medium for mineral extraction due to the poor solubility of minerals in it [64]. During the extraction of phenolic compounds, mineral extraction can potentially be enhanced through the process of complexation. This process implicates the formation of a stable complex between two or more molecules, typically through noncovalent bonds, in which a ligand bonds to a metal ion. This bonding alters the physical and chemical properties of the newly created complex [65]. Indeed, the interactions between metals and phenolic compounds (particularly flavonoids, such as quercetin and kaempferol) are well documented in the literature [66,67,68,69]. While multiple mechanisms may exist for their interaction, in the case of flavonoids, it is believed that they primarily interact through their carbonyl (C=O) and hydroxyl (-OH) groups, facilitating chelation with various metals [67]. Therefore, during the obtainment of the phenolic compounds from OP, stable complexes may form between phenolic compounds and metals present in the OP, increasing their solubility in ethanol and, consequently, leading to the presence of metals in the OP extract in the form of complexes.

### 3.2. Microparticles Production and Characterisation

Onion peel extract-loaded microparticles were produced using a water-in-oil-in-water (w/o/w) double emulsion solvent evaporation technique. The microparticles were produced with a theoretical loading of 10%. Results regarding the microencapsulation production yield (PY), encapsulation efficiency (EE), actual loading content (ALC), mean particle size (PS) and polydispersity index (PDI) are expressed in Table 2.

The analysis of Table 2 indicates that the PY was approximately 78%. This implies that the combination of ethyl cellulose and OP extract results in 78% of microparticles and in a total loss of 22%. A literature study employing the same technique for the microencapsulation of hydroxytyrosol revealed PY within 72–82%, indicating that the achieved value is within the literature range [29]. Regarding the EE, this parameter measures the amount of extract that was successfully embedded within the polymer. It can be concluded that the EE was between 88% and 93%, indicating that the OP extract was efficiently entrapped in ethyl cellulose microparticles. The achieved values are within the literature values for the use of double emulsion with ethyl cellulose [26,27]. Moreover, double emulsion achieved similar and superior EEs to spray-drying and freeze-drying, with values within 75–89% and 58–94%, respectively [70,71,72,73]. It can be concluded that this technique is effective to microencapsulate OP extract, resulting in elevated EE. Considering the ALC, it is noticeable that the average value was 6.0%, meaning that the actual amount of OP extract per gram of microparticles is 60 ± 1.5 mg_OP extract_·g_microparticles_^−1^. Results reveal a 40% decrease compared to the theoretical loading. A decrease in ALC was anticipated, as typically a fraction of the extract is lost to the aqueous phase [29]. Nevertheless, this decrease is inferior to the one observed in the literature, suggesting that the extract was successfully microencapsulated [26,27].

Regarding particle size (PS), this variable ranged from 4 μm to 31.8 μm, with an average diameter of 17.9 μm. The results reveal that the PS is within the desired range of micrometres, since it avoids the presence of nanoparticles that can infiltrate the bloodstream, which is unsought. The mean average size is within the typical literature values for OP extract-loaded microparticles [26,27,72,74,75]. Considering the particle size distribution (differential volume versus particle size), the microparticle formulation exhibited a unimodal PS distribution. Moreover, considering the PDI, it can be concluded that the sample is polydisperse, as the achieved value was 2.51. It has been observed that encapsulation of phenolic extracts using the double emulsion solvent evaporation technique tends to yield polydisperse microparticles [26,76]. The wide variety of bioactive compounds embedded in the microparticles can likely affect the PS and, consequently, impact the PS distribution of the sample [76]. The polydispersity of the microparticles significantly affects the uniformity of the powder, which, in turn, may influence the homogeneity of the fresh cheese after the fortification process and the distribution of microparticles within the matrix. Moreover, this phenomenon may also impact the release kinetics of the compounds from the particles, as the release may not be uniform over time.

### 3.3. Inclusion of the Phenolic Extract in the Microparticles

**Figure 1:**
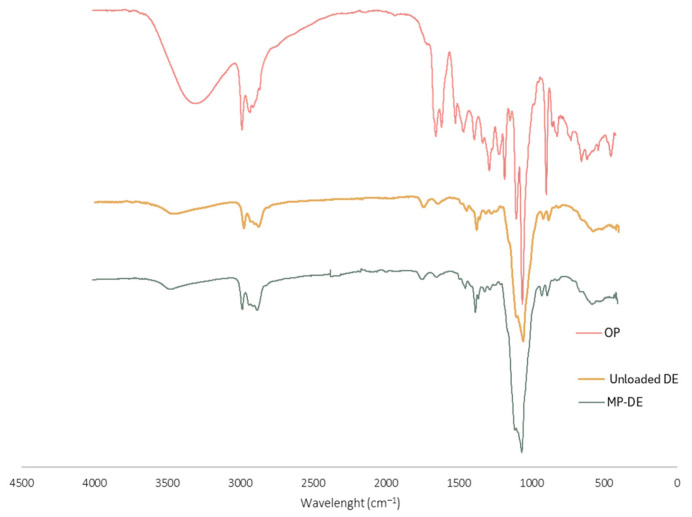
FTIR-ATR spectrum of different samples: unloaded microparticles, onion peel extract (OP) and onion peel extract-loaded microparticles (MP).

To understand if the phenolic extract from OP was successfully entrapped within the ethyl cellulose polymeric matrix, the FTIR-ATR technique was performed on the OP extract, polymer-only microparticles and OP extract-loaded microparticles. The achieved results are displayed in Figure 1.

Analysing the OP extract spectrum, there is an intense band at around 1700 cm^−1^, typically associated with the double C=O bond in saturated ketones present in phenolic compounds, including quercetin and kaempferol. Moreover, between 3000–3500 cm^−1^, there is a broad, intense peak related to the O-H stretching vibrations, indicating the presence of hydroxyl groups that are typical of phenolic compounds. Additionally, the spectrum exhibits two bands between 2900 and 3000 cm^−1^, which are associated with the aromatic and aliphatic C-H stretching vibrational mode. The bands at 1440 cm^−1^ and 1600 cm^−1^ relate to the double bond of the aromatic C=C, while the bands in the region between 1000 and 1300 cm^−1^ can be attributed to the C-O bonds of the different phenolics.

The spectrum of the polymer-only microparticles (unloaded) revealed two characteristic bands at approximately 2800–3000 cm^−1^, related to C-H stretching typical of alkane groups present in ethyl cellulose. Furthermore, the presence of a band at around 3500 cm^−1^, typical of O-H stretching vibration bonds, is present in the polymer due to its ethylation degree of 47%. The bands related to the bending vibration of -CH_3_ groups are also present in the spectrum at around 1450 cm^−1^. The most intense band of the spectrum is at around 1000 cm^−1^, which is attributed to the C-O stretching bonds.

Comparing the spectrum of the OP extract-loaded microparticles with the unloaded microparticles, it can be observed that there is high similarity between both. Additionally, it appears that the typical bands of the free OP extract disappeared in the microparticles spectrum, indicating that the extract was successfully encapsulated. This may also result from the microparticles’ low theoretical loading (10%). Nonetheless, results suggest that the OP was effectively entrapped within the ethyl cellulose network.

**Figure 2:**
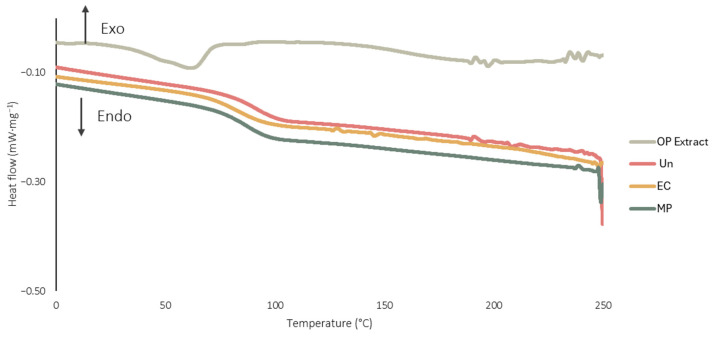
Differential Scanning Calorimetry thermograms of different samples: OP extract; OP extract-loaded microparticles (MP), unloaded microparticles (Un) and ethyl cellulose (EC).

To further evaluate the inclusion of the OP extract within the EC network a DSC analysis was carried out. The DSC thermograms of the OP extract, OP extract-loaded microparticles, unloaded microparticles and ethyl cellulose are displayed in Figure 2.

OP extract DSC thermogram exhibited an endothermic peak corresponding to the melting point of 67.6 °C (inflexion temperature). When analysing both unloaded microparticles and microparticles loaded with OP extract, a broad endothermic event is observed. Specifically, the temperature ranges are from 78.9 °C to 100.9 °C for the unloaded microparticles and from 74.4 °C to 96.9 °C for the extract-loaded microparticles, with peaks recorded at 90.8 °C and 86.8 °C, respectively. The EC thermogram also displays a broad peak between 68.9 °C and 95.8 °C, with a peak at 81.5 °C, characteristic of the glass transition for amorphous polymers, as is the case of EC [29]. Importantly, the DSC thermogram of the OP extract-loaded microparticles does not exhibit the typical melting peak associated with the extract; instead, it resembles the thermogram of pure EC. These findings suggest that the OP extract has undergone structural alterations, confirming its successful microencapsulation within the ethyl cellulose matrix [29,77]. Moreover, the transition temperature of the EC exhibits slight variations when comparing unloaded and loaded microparticle formulations. In polymer-based microencapsulation systems, such variations are typically attributed to interactions between the active ingredient and the polymer matrix [29]. This observation suggests that significant interactions occur between the OP extract and EC. The DSC results further reinforce this interpretation, as the absence of a distinct peak and a separate transition related to the extract in the thermogram of the loaded microparticles indicates that the extract has been effectively encapsulated within the polymer matrix.

### 3.4. Evaluation of the Morphology

**Figure 3:**
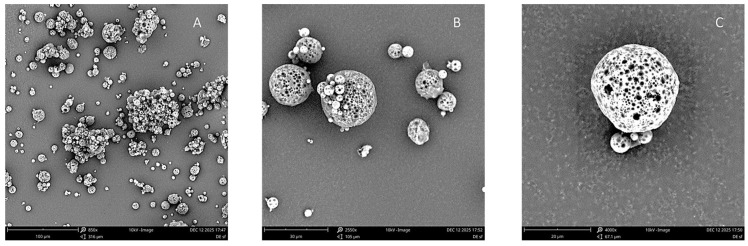
Microphotographs of OP extract-loaded microparticles at three different magnifications: 850× (**A**), 2550× (**B**) and 4000× (**C**).

Microphotographs of the achieved microparticles are presented in Figure 3, at three different magnifications (850, 2550, and 4000 times).

Analysing the microphotographs, it can be observed that the produced microparticles are spherical with a smooth surface, lacking cracks and/or fissures and without irregular bumps. Moreover, the microparticles are porous. Results indicate that the solvent evaporation was adequate [78]. SEM microphotographs reveal an agglomeration of microparticles, as shown in Figure 2A, which contributes to the wide range of particle sizes observed in the sample. This result corroborates the conclusion achieved from the mean PS analysis, that the microparticles are polydisperse and heterogeneous. Literature reports a tendency for the formation of clusters in microparticle formulations using ethyl cellulose and the double emulsion technique [19,79].

### 3.5. Physicochemical Characterisation of the Produced Cheeses

**Figure 4:**
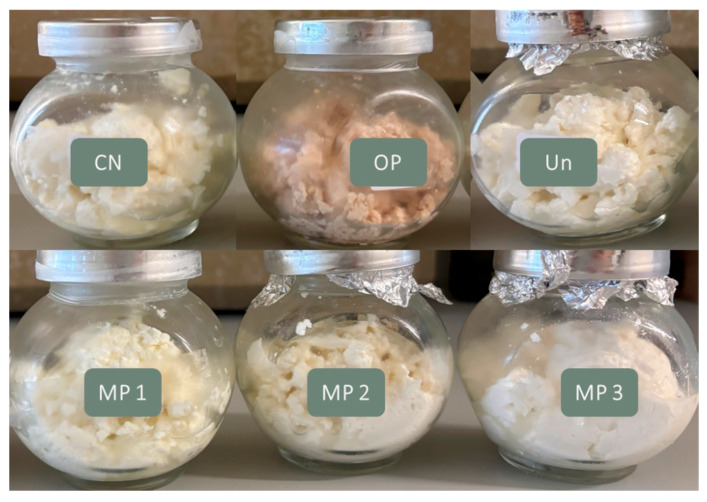
Produced fresh cheeses. NC: Negative control; OP: Cheese with 1 g∙L^−1^ of OP extract; Un: Cheese with 1 g∙L^−1^ of unloaded microparticles; MP 1: Cheese with 1 g∙L^−1^ of OP extract-loaded microparticles; MP 2: Cheese with 2.5 g∙L^−1^ of OP extract-loaded microparticles; MP 3: Cheese with 5 g∙L^−1^ of OP extract-loaded microparticles.

The present study aimed to evaluate the ability of OP extract, free and microencapsulated, to improve the nutritional value of fresh cheese. Consequently, six cheeses were produced: NC—Negative control (without additives), OP—fresh cheese with 1 g∙L^−1^ of OP extract, Un– fresh cheese with 1 g∙L^−1^ of unloaded microparticles, MP 1—fresh cheese with 1 g∙L^−1^ of OP extract-loaded microparticles, MP 2—fresh cheese with 2.5 g∙L^−1^ of OP extract-loaded microparticles, MP 3—fresh cheese with 5 g∙L^−1^ of OP extract-loaded microparticles. The samples produced are displayed in Figure 4. Direct observation indicates that the addition of the free OP extract results in a significant alteration in the colour of the cheese, shifting towards a more pinkish or reddish tone. Although there is a considerable trend towards innovation in cheese varieties and the development of cheeses with different colours, consumers continue to favour cheeses with their traditional colours. In contrast, the incorporation of the microencapsulated OP extract significantly minimises these colour changes, resulting in fortified cheeses that resemble the appearance of the NC sample. This observation is positive, suggesting that the use of the microencapsulated extract successfully maintains the product’s colour characteristics, addressing the limitation associated with the use of the free extract.

**Figure 5:**
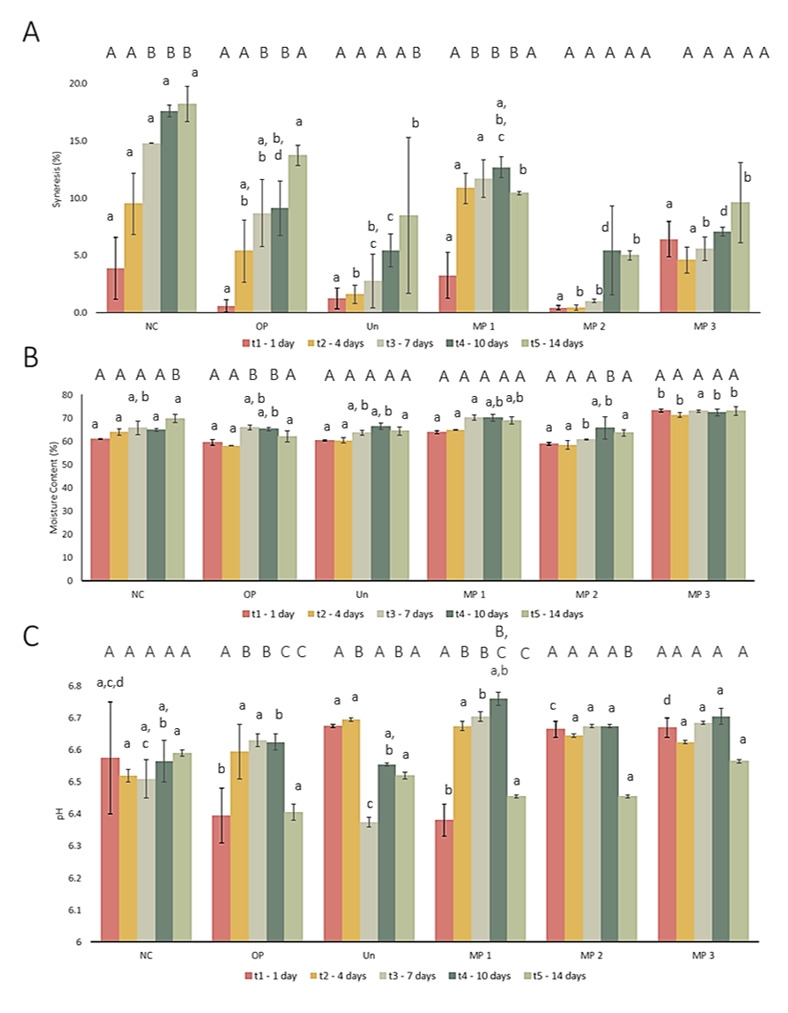
Physicochemical analysis of the produced cheeses, for the five analysis time points: (**A**) Syneresis; (**B**) Water content; (**C**) pH. NC: Negative control; OP: Cheese with 1 g∙L^−1^ of OP extract; Un: Cheese with 1 g∙L^−1^ of unloaded microparticles; MP 1: Cheese with 1 g∙L^−1^ of OP extract-loaded microparticles; MP 2: Cheese with 2.5 g∙L^−1^ of OP extract-loaded microparticles; MP 3: Cheese with 5 g∙L^−1^ of OP extract-loaded microparticles. The results are expressed as means ± standard deviations of *n* = 3 independent measurements. The different capital letters represent significantly different values (*p* < 0.05) for the same sample, at different times. Different small letters represent significantly different values (*p* < 0.05) between samples at the same time point.

The physicochemical properties of the samples, such as syneresis, moisture content, and pH, were analysed to evaluate the impact of both free and microencapsulated OP extract on the cheese. The results achieved are displayed in Figure 5.

Syneresis refers to the separation of serum (whey) from cheese, occurring due to structural changes in the product during storage. Analysis of the results shows that this phenomenon increased over time in all samples. Notably, the sample with the highest level of syneresis was the negative control (NC), followed by the cheese fortified with extract (OP) and the cheese containing polymer-only microparticles (Unloaded). Additionally, microparticles effectively reduced syneresis, with a particularly significant decrease observed in samples MP 2 and MP 3, which contained more microparticles. The findings indicate that microencapsulation significantly mitigated whey release compared to the free extract. This underscores the critical role of microencapsulation in effectively reducing cheese syneresis. Although a decrease in this variable was noted over time, results can be considered positive as the incorporation of the OP extract improved cheese capacity to maintain the structure of the casein network and, consequently, retain whey. In fact, literature reports have stated that phenolic compounds can interact with proteins, including casein, forming stable complexes that prevent protein rearrangement during storage, preserving the casein network [31,80]. Moreover, the ability of phenolic compounds to bond with water can also improve water retention, decreasing the amount of whey released and, consequently, syneresis.

Considering moisture content, this variable was analysed during storage to determine whether the addition of free and microencapsulated OP extract influenced the characteristics of fresh cheese. Results reveal that the moisture content of the samples was constant during storage, indicating that the observed changes were not significant. All samples exhibited moisture content values within the range of 60–70%, with sample MP 3 displaying the highest values. These values are within the typical range for this type of cheese, between 60% and 80%, corresponding to its characterisation as a semi-soft to soft cheese [81]. Moreover, results demonstrate that OP-fortified samples did not exhibit a significant difference (*p* > 0.05) compared to NC, except for sample MP 3, as shown by the statistical analysis. It is observable that the moisture content had a slight decrease for samples containing microparticles (Unloaded, MP 1 and MP 2). Additionally, the literature reports that the incorporation of phenolic extracts into cheese may contribute to an increase in the moisture content of the sample; however, in the present study, this increase was not significant, except for sample MP 3. This indicates that an increase in the amount of microparticles added to the fresh cheese might influence the moisture content of the sample [26,82].

The pH level is a critical parameter in the quality assessment of fresh cheese, as it influences not only the cheese’s structural integrity but also its susceptibility to microbial and bacterial growth, and its potential for spoilage. Therefore, pH measurements must be conducted periodically to ensure product safety and quality. The analysis revealed that the samples’ pH ranged from 6.3 to 6.8 during the study period. The achieved values are within the desired range for fresh cheese produced with cow’s milk, typically between 6.2 and 6.8 [26,83]. Notably, the pH values for the samples fortified with OP extract, both free and microencapsulated, were similar to those of NC, except for time t_1_. This indicates that incorporation of the extract did not significantly impact pH levels. Additionally, while some pH fluctuations were noted during the storage, all values remained within the acceptable range. Moreover, incorporating microparticles with ethyl cellulose increases the pH value of the samples. In fact, this polymer is considered neutral, which can potentially increase the pH, as reported in the literature [26]. Results reveal a small decrease in pH with time, which can increase syneresis, as previously observed, due to rearrangements within the casein protein-protein bonds [84,85].

### 3.6. Evaluation of the General and Mineral Composition of the Produced Fresh Cheeses

**Figure 6:**
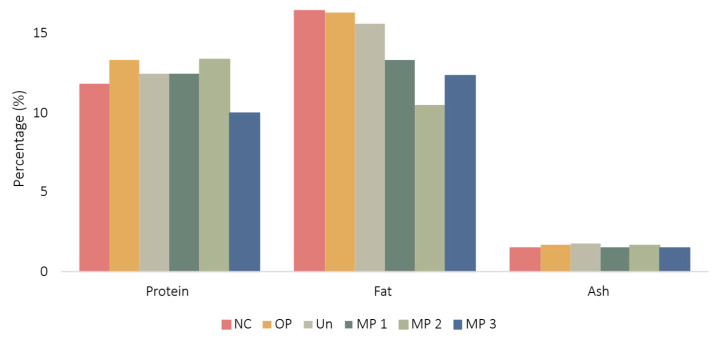
Protein, fat and ash content, in percentage, of the produced cheeses. NC: Negative control; OP: Cheese with 1 g∙L^−1^ of OP extract; Un: Cheese with 1 g∙L^−1^ of unloaded microparticles; MP 1: Cheese with 1 g∙L^−1^ of OP extract-loaded microparticles; MP 2: Cheese with 2.5 g∙L^−1^ of OP extract-loaded microparticles; MP 3: Cheese with 5 g∙L^−1^ of OP extract-loaded microparticles.

Fresh cheese is characterised as cheese that does not undergo maturation, resulting directly from the coagulation of milk following heating and the incorporation of rennet. The results of the proximate composition analysis of the produced cheeses for protein, fat and ash content are displayed in Figure 6.

From Figure 6, it can be observed that the proximate composition was considerably similar for the six cheeses, except for the fat content. Considering the ash content, the values were extremely similar between samples, indicating that the inclusion of the extract did not impact this parameter. It is noticeable that the incorporation of the extract, both free and microencapsulated, increased the protein content of the samples, with samples MP 2 and OP exhibiting the highest values of 13.4 and 13.3%, respectively. Samples Un and MP 1 display similar values, considering protein content increase, indicating that the polymer might affect this property and that a concentration of 1 g∙L^−1^ of OP extract-loaded microparticles may not be sufficient to have a significant impact on this variable. Considering sample MP 3, it was expected that this sample exhibited higher protein content compared to formulations MP 1 and MP 2; however, this was not verified. This observation indicates that the distribution of microparticles within the cheese matrix may be heterogeneous or that the release of OP extract from the microparticles is minimal. This phenomenon could be attributed to the polydispersity of microparticles, which may affect extract release. The small increase in protein content for extract-fortified samples can relate to the interaction between phenolic compounds and milk proteins, which can result in cross-linking or precipitation, promoting the fixation of soluble proteins within the para-casein network or the development of small protein aggregates, increasing protein retention and, consequently, protein content [82,86].

Regarding fat content, the incorporation of the MP significantly decreased this variable. Indeed, sample NC displays the highest fat content of 16.5%, followed by OP with 16.3%. Samples fortified with microencapsulated OP-extract, MP 1, MP 2 and MP 3, displayed values of 13.3, 10.5 and 12.4% of fat content, respectively. Despite the positive result, from a nutritional perspective, the literature has not described phenolic compounds’ capacity to reduce fat content. Therefore, the observed differences may be related to sample heterogeneity or to possible interactions between ethyl cellulose and fats, since this polymer is commonly used to microencapsulate oils and hydrophobic compounds, including lipids [19,87,88]. Moreover, it has been documented that ethyl cellulose can interact with oils, thereby contributing to their stabilisation [89]. However, the existing literature does not thoroughly explore potential interactions or limitations of ethyl cellulose with the Folch method for lipid extraction. This method relies on a solvent mixture that must effectively penetrate the sample matrix to ensure complete lipid extraction [45,90]. Consequently, the potential entrapment of lipids within the ethyl cellulose network may hinder the solvent’s ability to penetrate the sample effectively. As a result, the recovery of lipids is weakened in samples containing ethyl cellulose microparticles when compared to those without particles.

Minerals are essential compounds for the human body, as they serve a variety of biological functions, playing key roles in balancing the acid-base conditions of the human body and osmotic pressure [91]; moreover, they can maintain haemoglobin levels and are crucial for neuromuscular transmission, enzymatic activity, structural roles (bones, teeth, cell membranes) and muscle contraction [91,92,93]. To understand if the inclusion of the extract, both free and microencapsulated, affected the mineral composition of the cheese, ICP analysis was performed on the samples, and the results are in Table 3.

Results reveal that the main minerals present in the cheese were sodium (Na), followed by potassium (K), calcium (Ca) and magnesium (Mg). Sodium may be detected in superior concentrations due to the incorporation of salt during fresh cheese production. In fact, the literature reports that calcium is a main mineral in cheese, along with phosphorus, potassium and sodium [94]. The sample with the highest Ca content is MP 1, at 5.34 mg∙g^−1^, followed by MP 2 and NC. Results indicate that samples OP and MP 3 display inferior content of this mineral. Nevertheless, the achieved values are within the literature ones for soft cheeses produced from cow milk, ranging from 2.3 to 5.5 mg∙g^−1^ [95]. Considering the K content, the inclusion of the microencapsulated OP extract increased the concentration of this mineral. Regarding the Na concentration, the inclusion of the extract increased the sodium concentration, especially for the free extract. Focusing on the Mg content, the results display less variability compared to K and Na, with samples MP 1 and MP 2 exhibiting slightly higher values with concentrations of 3.93 and 3.44 mg∙g^−1^, respectively.

It was anticipated that MP 3 exhibited superior mineral concentration compared to MP 1 and MP 2, considering that the amount of microparticles and, consequently, of extract was considerably superior; however, the opposite is observed. Results suggest that the release of the compounds and minerals from the microparticles may not be uniform, meaning that a superior microparticle concentration does not ensure a superior concentration of bioactive compounds in the sample. A study focused on Cheddar cheese fortification with microencapsulated ferrous sulphate indicated that smaller particle sizes are associated with significantly higher mineral content compared to larger ones [96]. As previously observed, the produced microparticles were polydisperse, suggesting the presence of particles of varying sizes within the cheese matrix. This variability can substantially impact the bioavailability and kinetic release of the OP extract into the cheese. Additionally, the interactions between smaller microparticles and the lipids in cheese may be enhanced by an increase in surface area, while interactions with larger particles may be diminished or minor [96].

These findings reveal that the incorporation of phenolic extract into fresh cheese increases certain minerals, particularly Ca, K, Mg and Na, especially for the encapsulated extract. Existing literature suggests that phenolic compounds can interact with various minerals in food matrices and chelate specific minerals, such as Ca^2+^, potentially increasing their levels within the food product [97,98,99]. Furthermore, analysing the effect of the sample Un, it is noticeable that ethyl cellulose increases the content of some minerals, mainly K and Na, in comparison to sample NC. This suggests that the polymer may retain some minerals during fresh cheese production, enhancing their presence on the final product. Notably, the addition of microencapsulated extract mitigates the decrease in mineral content observed in fresh cheese supplemented with free extract, particularly in formulations MP 1 and MP 2. This suggests that microencapsulation may serve as an effective strategy to address the limitations associated with the direct inclusion of free OP extract in fresh cheese.

### 3.7. Impact on the Total Phenolic Content (TPC) and DPPH Scavenging Capacity of the Fresh Cheese

**Figure 7:**
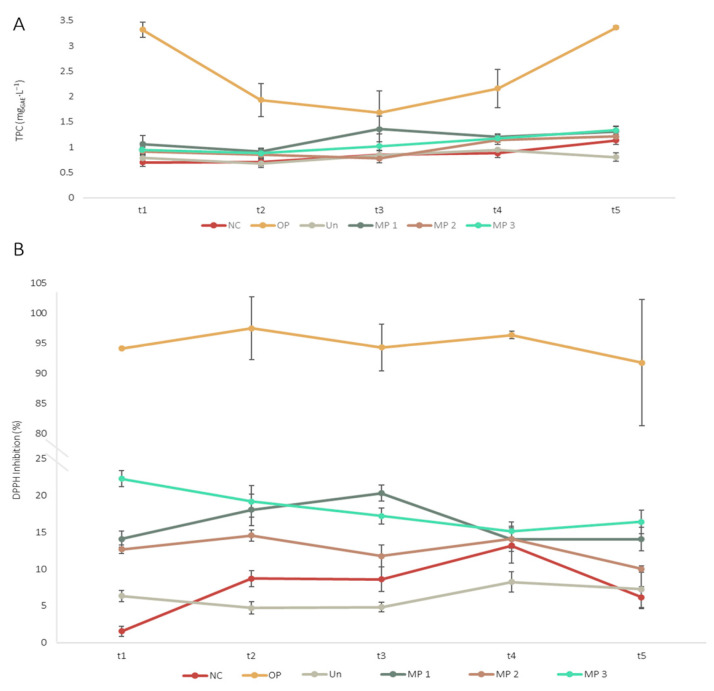
Variation in the TPC (**A**) and DPPH inhibition (**B**) for the produced cheeses, during storage. NC: Negative control; OP: Cheese with 1 g∙L^−1^ of OP extract; Un: Cheese with 1 g∙L^−1^ of unloaded microparticles; MP 1: Cheese with 1 g∙L^−1^ of OP extract-loaded microparticles; MP 2: Cheese with 2.5 g∙L^−1^ of OP extract-loaded microparticles; MP 3: Cheese with 5 g∙L^−1^ of OP extract-loaded microparticles. The results are expressed as means ± standard deviations of three independent measurements obtained from the same sample.

To understand the effect of adding the OP extract, free and microencapsulated, to the fresh cheese, the total phenolic content (TPC) and the DPPH scavenging capacity of the samples were evaluated. Results are presented in Figure 7.

Upon analysing the results for the TPC, the cheese fortified with free OP extract exhibited superior values, indicating a high content of phenolic compounds. Moreover, the sample containing ethyl cellulose only microparticles (Un) performed similarly to the negative control (NC) sample, with the lowest TPC values. This result implies that the presence of the polymer does not influence this variable. Additionally, it is noticeable that samples containing microparticles (MP 1, MP 2 and MP 3) revealed a similar behaviour regarding TPC values, indicating that the amount of microparticles added did not significantly alter the TPC of the samples. Comparing the behaviour of the fortified samples with OP microparticles with sample OP, the TPC values of the latter are considerably superior; however, the TPC values for microparticle fortified samples increase during storage, demonstrating the effect of the controlled release of the extract from the polymer and, consequently, its protection against oxidation. Regarding the sample OP, the TPC decreases from t_1_ to t_3_, followed by an increase. This can result from the degradation of phenolic compounds due to exposure to light and oxygen, leading to their oxidation and reducing their biological activity. Afterwards, there is an increase in TPC, which is observable for all samples. This behaviour might result from microbial activity that leads to changes in protein structure, the release of small-chain peptides and amino acids, and the release of the phenolic compounds from the cheese network [100,101,102]. The presence of these compounds, along with other components, such as vitamin C and E, which possess antioxidant capacity, can interfere with the *Folin–Ciocalteu* reagent due to its lack of selectivity, resulting in an increase in the TPC value over time. Nevertheless, the obtained results demonstrate the potential of OP extract, both free and microencapsulated, to improve the TPC of fresh cheese.

The results indicate a similar behaviour between the DPPH inhibition outcomes and the TPC values. For the sample OP, the scavenging capacity is approximately 95% at the start of the storage period (t_1_), decreasing to 90% by the end of the study (t_5_), indicating potential degradation of the antioxidant compounds. Although a decline in DPPH inhibition was observed, this change did not reach statistical significance. This output corroborates the TPC results, which suggest that this sample may be rich in phenolic compounds that can donate hydrogen atoms, stabilising free radicals, including the DPPH one. Similar behaviour was observed for samples MP 1, MP 2 and MP 3, with fresh cheese MP 3 exhibiting superior DPPH inhibition. Despite some variations in the DPPH inhibition, the results indicate that the amount of microparticles added did not significantly impact this parameter, since the results at time point t_5_ are similar for the three samples, with MP 3 exhibiting superior values, followed by MP 1 and MP 2. This outcome may result from the controlled release of the extract from the microparticles, which allows a constant concentration of phenolic compounds within the cheese matrix. Moreover, the results reveal an increase in the inhibitory action towards the DPPH radical of the fortified samples compared to the negative control. This suggests that both the free and microencapsulated OP extract were successfully incorporated into the cheese and retained within the matrix. In fact, the literature reports that hydrophobic compounds (such as some phenolics, including quercetin, and ethyl cellulose) are embedded in the matrix. In contrast, hydrophilic ones are lost with the whey [86]. Furthermore, the sample NC demonstrates notable antioxidant capacity, attributed to the presence of various antioxidant compounds, including peptides, fatty acids, and essential vitamins, such as A, C, and E, which play a role in inhibiting DPPH. Nonetheless, the results demonstrate that the incorporation of the OP extract, both free and microencapsulated, improves the DPPH scavenging and enhances the antioxidant potential of the fresh cheese.

The findings of this study indicate that although the incorporation of varying amounts of OP extract-loaded microparticles improved the TPC and DPPH inhibition levels among the samples, it did not result in a significant difference. However, it is noteworthy that the theoretical loading of the microparticles was 10%, meaning that the amount of OP extract present in the formulations fortified with particles is considerably inferior to the quantity of extract added to the OP sample. To further understand the potential effects of microparticle loading on these variables, it would be interesting to explore additional theoretical loadings of 15% and 20%. This investigation could determine whether these changes might lead to results comparable to those observed in cheese fortified with free OP extract.

## 4. Conclusions

To address the adverse impacts of onion peels, a major agro-industrial by-product, a phenolic-rich extract was obtained and incorporated into fresh cheese, in both free and microencapsulated forms, to enhance its nutritional composition. The extract was obtained via Soxhlet extraction with ethanol as the extraction solvent. Extract characterisation revealed a total phenolic content (TPC) of 513 mg_GAE_∙g_extract_^−1^, while antioxidant assays uncovered that the extract was more efficient at scavenging the ABTS radical than the DPPH one; however, it demonstrated strong antioxidant capacity towards both radicals, as observed by their IC_50_ of 9.5 mg_Extract_∙L^−1^ for ABTS and 36.1 mg_Extract_∙L^−1^ for DPPH. Moreover, the extract demonstrated activity towards biological radicals, with an IC_50_ of 91.5 mg_Extract_∙L^−1^ towards the superoxide radical and of 282 mg_Extract_∙L^−1^ towards the nitric oxide radical. The OP extract demonstrated inhibitory action towards α-amylase and β-glucosidase with inhibitions of 71% and 82%, respectively, which may contribute to potential anti-diabetic properties. The HPLC analysis identified quercetin as the predominant phenolic compound present in the extract at a concentration of 58 mg∙g_extract_^−1^. Mineral analysis indicated that potassium was the main mineral found at a concentration of 374 µg∙g_extract_^−1^.

The OP extract was microencapsulated using the w/o/w double emulsion solvent evaporation technique to improve its stability. The production yield was 78%, while the encapsulation efficiency and actual loading content were 91% and 6%, respectively. The average particle size was 17.9 µm, and the sample was polydisperse. SEM analysis further corroborated these findings, illustrating the formation of aggregated microparticles with a smooth, porous surface morphology.

Fresh cheese fortification with the free OP extract originated colour changes, which were overcome by adding the extract in its microencapsulated form. Additionally, incorporating the extract reduced cheese syneresis, a positive outcome, while maintaining typical moisture content (60–80%) and pH (6.3–6.8). Proximate composition analysis revealed that the samples exhibited similar protein and ash content, whereas the fat content was lower for extract-fortified samples. The mineral analysis concluded that the inclusion of the free OP extract decreased the cheese mineral content. This limitation was overcome by using microencapsulated OP extract. It was found that the inclusion of the free OP extract significantly increased the TPC and DPPH scavenging capacity of the cheese. Samples fortified with microparticles exhibited an increase in these properties during storage, which may be related to the controlled release of the extract from the particles.

Overall, the results of the present study demonstrate that the incorporation of OP extract, both in free and microencapsulated forms, enhanced the antioxidant profile of the fresh cheese. This study presents a promising strategy for the valorisation of OP by incorporating phenolic-rich extracts into fresh cheese. The findings have significant implications for practical industrial applications, as enhancing the antioxidant content of the product may facilitate the development of a value-added food with extended shelf life. This approach not only enhances the nutritional composition of fresh cheese but also aligns with the growing consumer demand for products that offer functional health benefits.

## Figures and Tables

**Table 1:** Biological characterisation of the phenolic-rich extract from onion peels. Chemical and mineral composition results.

TPC (mg_GAE_∙g_extract_^−1^)		513 ± 36.8
**ABTS**	IC_50_ (mg_Extract_∙L^−1^)	9.5 ± 0.3
	TE (mg_Trolox_∙g_extract_^−1^)	307 ± 10.5
**DPPH**	IC_50_ (mg_Extract_∙L^−1^)	36.1 ± 0.7
	TE (mg_Trolox_∙g_extract_^−1^)	222 ± 4.6
**Superoxide**	Inhibition at c = 0.5 mg∙mL^−1^ (%) IC_50_ (mg_Extract_∙L^−1^)	98.6 ± 0.8
		91.5 ± 2.1
**Nitric oxide**	Inhibition at c = 0.5 mg∙mL^−1^ (%) IC_50_ (mg_Extract_∙L^−1^)	41.9 ± 1.7 282 ± 5.4
**α-amylase inhibition** (%)		70.8 ± 3.0
**β-glucosidase inhibition** (%)		81.8 ± 5.6
**HPLC Analysis (mg_Compound_ ∙ g^−1^)**		
Epicatechin		8.9 ± 0.2
Kaempferol		4.0 ± 0.9
Quercetin		58.0 ± 0.3
Resveratrol		7.5 ± 0.3
**ICP Analysis** (µg∙g^−1^)		
**Mineral**	**OP Powder**	**OP Extract**
Al	423 ± 43.9	31.0 ± 1.0
B	56.1 ± 16.9	N.D.
Ca	2.1 × 10^4^ ± 1.7 × 10^3^	187 ± 37.6
Fe	246 ± 4.9	23.4 ± 3.2
K	7.3 × 10^3^ ± 1.5 × 10^2^	374 ± 3.1
Li	25.8 ± 0.8	2.8 ± 0.2
Mg	2.7 × 10^3^ ± 7.9 × 10^2^	43.6 ± 0.9
Na	2.8 × 10^3^ ± 8.5 × 10^2^	254 ± 22.9
Sr	23.7 ± 3.1	N.D.

ABTS: 2,2′-azinobis(3-ethylbenzothiazoline-6-sulfonic acid); DPPH: 2,2-diphenyl-1-picrylhydrazyl; GAE: Gallic acid equivalents; IC_50_: Needed concentration to inhibit 50% of the radicals; N.D.: Non detected; OP: Onion peels; TE: Trolox equivalents; TPC: Total phenolic content.

**Table 2:** Main physicochemical characteristics of onion peel extract-loaded microparticles.

PY (%)	EE (%)	ALC (%)	PS (µm)	PDI
77.5 ± 6.6	90.5 ± 2.3	6.0 ± 0.2	17.9 ± 13.9	2.51

ALC: Actual loading content; EE: Encapsulation efficiency; PDI: Polydispersity index; PS: Particle size; PY: Production yield.

**Table 3:** Mineral composition of the produced fresh cheeses.

		Sample					
		NC	OP	Un	MP 1	MP 2	MP 3
Mineral (mg ∙ g^−1^)	Ba	0.04 ± 0.03 ^a^	0.03 ± 0.02 ^a^	0.01 ± 0.00 ^a^	0.21 ± 0.00	0.05 ± 0.00 ^b^	0.02 ± 0.00 ^a^
	Ca	4.25 ± 0.04 ^a^	2.15 ± 0.10 ^a^	3.84 ± 0.01 ^a^	5.34 ± 0.01 ^a^	4.87 ± 0.01 ^a^	3.46 ± 0.01 ^a^
	K	5.21 ± 0.65 ^a^	3.40 ± 9.40 ^a,b^	5.81 ± 0.38 ^a^	6.30 ± 0.10 ^a,c^	7.10 ± 0.02 ^a^	6.28 ± 0.09 ^a^
	Mg	3.20 ± 016 ^a^	1.74 ± 0.00 ^a^	2.65 ± 0.27 ^a^	3.93 ± 0.00 ^a^	3.84 ± 0.03 ^a^	2.83 ± 0.01 ^a^
	Na	13.9 ± 0.22 ^a^	22.9 ± 0.03 ^b^	18.41 ± 0.61 ^c^	19.30 ± 0.14 ^c^	20.92 ± 0.12 ^c^	21.10 ± 0.02 ^b,c^
	Sr	0.19 ± 0.06 ^a^	N.D.	0.14 ± 0.00 ^a^	0.17 ± 0.00 ^a^	0.15 ± 0.00 ^a^	0.09 ± 0.01 ^a^
	Zn	0.01 ± 0.00 ^a^	0.01 ± 0.04 ^a^	0.01 ± 0.00 ^a^	0.01 ± 0.00 ^a^	0.02 ± 0.00 ^a^	N.D.

NC: Negative control; OP: Cheese with 1 g∙L^−1^ of OP extract; Un: Cheese with 1 g∙L^−1^ of unloaded microparticles; MP 1: Cheese with 1 g∙L^−1^ of OP extract-loaded microparticles; MP 2: Cheese with 2.5 g∙L^−1^ of OP extract-loaded microparticles; MP 3: Cheese with 5 g∙L^−1^ of OP extract-loaded microparticles. The results are expressed as means ± standard deviations of three independent measurements obtained from the same sample. Different lowercase letters in each line represent a statistically significant difference between the results in each sample.

## Data Availability

The original contributions presented in the study are included in the article, further inquiries can be directed to the corresponding author.
